# SUMO modification system facilitates the exchange of histone variant H2A.Z-2 at DNA damage sites

**DOI:** 10.1080/19491034.2017.1395543

**Published:** 2017-12-14

**Authors:** Atsuhiko Fukuto, Masae Ikura, Tsuyoshi Ikura, Jiying Sun, Yasunori Horikoshi, Hiroki Shima, Kazuhiko Igarashi, Masayuki Kusakabe, Masahiko Harata, Naoki Horikoshi, Hitoshi Kurumizaka, Yoshiaki Kiuchi, Satoshi Tashiro

**Affiliations:** a Department of Cellular Biology, Research Institute for Radiation Biology and Medicine, Hiroshima University, Hiroshima, Japan; b Department of Ophthalmology and Visual Science, Graduate School of Biomedical Sciences, Hiroshima University, Hiroshima, Japan; c Laboratory of Chromatin Regulatory Network, Department of Mutagenesis, Radiation Biology Center, Kyoto University, Kyoto, Japan; d Department of Biochemistry, Tohoku University Graduate School of Medicine, Sendai, Miyagi, Japan; e Laboratory of Molecular Biology, Graduate School of Agricultural Science, Tohoku University, Sendai, Miyagi, Japan; f Laboratory of Structural Biology, Graduate School of Advanced Science and Engineering, Waseda University, Shinjukuku, Tokyo, Japan; g Present address; Department of Structural Biology, School of Medicine, Stanford University, Stanford, CA, USA

**Keywords:** H2A.Z-2, PIAS4, SUMO, histone variant, DNA damage

## Abstract

Histone exchange and histone post-translational modifications play important roles in the regulation of DNA metabolism, by re-organizing the chromatin configuration. We previously demonstrated that the histone variant H2A.Z-2 is rapidly exchanged at damaged sites after DNA double strand break induction in human cells. In yeast, the small ubiquitin-like modifier (SUMO) modification of H2A.Z is involved in the DNA damage response. However, whether the SUMO modification regulates the exchange of human H2A.Z-2 at DNA damage sites remains unclear. Here, we show that H2A.Z-2 is SUMOylated in a damage-dependent manner, and the SUMOylation of H2A.Z-2 is suppressed by the depletion of the SUMO E3 ligase, PIAS4. Moreover, PIAS4 depletion represses the incorporation and eviction of H2A.Z-2 at damaged sites. These findings demonstrate that the PIAS4-mediated SUMOylation regulates the exchange of H2A.Z-2 at DNA damage sites.

## Introduction

DNA double strand breaks (DSBs) are one of the most serious forms of DNA damage. DSBs can be lethal to a cell, and errors in the repair process lead to genomic instability and tumorigenesis. There are two major repair pathways for DSB repair, homologous recombination (HR) and non-homologous end joining (NHEJ).^1^ HR ensures accurate repair by using the undamaged sister chromatid or homologous chromosome as the template. Several lines of evidence suggested that higher-order chromatin structures are reorganized by post-translational protein modifications and/or histone protein exchange at damaged sites to facilitate DNA damage repair. The best-known example is the phosphorylation of the histone H2A variant H2AX, called γH2AX and a marker of DSBs, which triggers almost all DNA damage responses, including various chromatin dynamics for DSB repair.^2^ In budding yeast, the SWR1 chromatin-remodeling complex catalyzes the replacement of H2A with the H2A variant H2A.Z.^3^ The SWR1 complex-dependent incorporation of H2A.Z is required for DSB relocation to the nuclear periphery.^4^ In mammalian cells, the NuA4 complex promotes the rapid exchange of H2A for H2A.Z at DSBs, suggesting a role of H2A.Z in the regulation of DNA repair in human cells.^5^ However, the function of H2A.Z in the reorganization of damaged chromatin in human cells is still unclear.

H2A.Z is an evolutionarily well-conserved histone variant from yeast to humans.^6^ The H2A.Z protein levels are ∼10% of the total H2A complement. In mice, deletion of the H2A.Z gene leads to early embryonic lethality.^7^ The absence of H2A.Z in yeast increases the sensitivity to genotoxic agents.^8^ H2A.Z is highly expressed in progressive breast cancer, bladder cancer and malignant melanoma.^9-11^ While H2A.Z is associated preferentially with the promoters of repressed genes, its K14 acetylated form is enriched at the promoters of active genes.^12^ A single gene (HTZ1) encodes H2A.Z in budding yeast, and two genes have been identified in vertebrates. These were named H2A.Z-1 (previously H2A.Z) and H2A.Z-2 (previously H2A.F/Z or H2A.V).^13^ H2A.Z-2-deficient cells proliferate more slowly than H2A.Z-1-deficient cells.^14^ We previously reported that RAD51 focus formation, a hallmark of recombinational repair, was disturbed in *H2A.Z-2*-deficient cells but not in *H2A.Z-1*-deficient cells.^15^ We also found that H2A.Z-2 is exchanged at DSB sites immediately after the induction of DSBs.^15^ However, the means by which the exchange of H2A.Z-2 is facilitated at damaged sites still remain unclear.

Histones and their variants can be modified post-translationally, by acetylation, methylation, and phosphorylation.^16-18^ They also can be conjugated to small proteins, such as ubiquitin and small ubiquitin-like modifier (SUMO).^19,20^ SUMOylation is a post-translational modification involved in cell cycle progression, subcellular transport, transcription and DNA repair.^21^ Chromosome-wide RAD51 spreading and SUMOylated H2A.Z are required for the movement of persistent DSBs to the nuclear periphery in yeast.^22^ In mammalian cells, SUMO proteins accumulate at DSB sites by mechanisms requiring MDC1, 53BP1 and BRCA1. Furthermore, the SUMO E3-ligases PIAS1 and PIAS4 accumulate at DSB sites to promote DNA repair by homologous recombination.^23^ We reported that the RAD51 accumulation at damaged sites is dependent on its SUMO interacting motif (SIM).^24^ However, it remains to be clarified whether SUMOylation is involved in the regulation of the exchange of human H2A.Z-2 at damaged sites.

Here, we showed that H2A.Z-2 is SUMOylated by PIAS4 in a damage-dependent manner in human cells. The depletion of PIAS4, but not PIAS1, significantly repressed the increase of the H2A.Z-2 mobility at sites containing DNA damage after microirradiation. These findings suggest that the SUMOylation of H2A.Z-2 is required for its exchange at sites of DNA damage.

## Results

**Figure 1.**
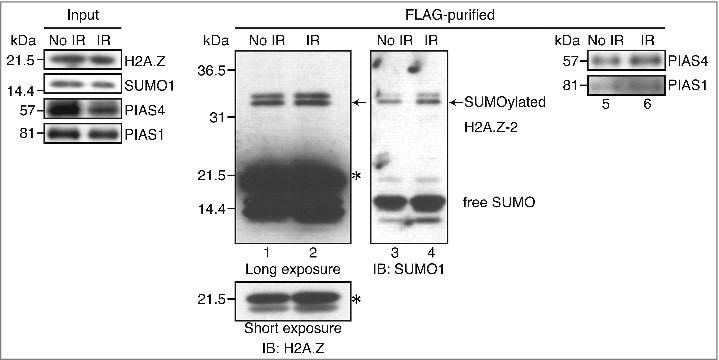
The H2A.Z-2 complex, purified from the nuclear soluble fraction of HeLa cells, was subjected to immunoblot analyses using anti-H2A.Z (lanes 1 and 2), anti-SUMO1 (lanes 3 and 4), anti-PIAS4 (lanes 5 and 6) and anti-PIAS1 (lanes 5 and 6) antibodies. DNA damage was induced by 10 Gy IR, followed by a 10-minute recovery. The arrows indicate SUMOylated H2A.Z-2 and the asterisks indicate unmodified H2A.Z-2. Nuclear extracts are used as the input.

To assess whether human H2A.Z-2 is SUMOylated after the induction of DNA damage, we established HeLa cells stably expressing C-terminally FLAG-HA-tagged H2A.Z-2. The histone H2A.Z-2 proteins were purified from the nuclear extracts of these cells before and after ionizing radiation (IR), as previously described.^25^ We subsequently performed the immunoblotting analysis using an anti-H2A.Z antibody, to confirm the presence of H2A.Z-2 proteins in the purified complex, and observed slowly migrating bands (arrows, Fig. 1) in addition to those with the expected size around 21.5 kDa, suggesting the posttranslational modification of H2A.Z-2. These slowly migrating bands were also detected by the immunoblotting using an antibody against SUMO1, and considering their molecular weight, these results led to the conclusion that they are SUMOylated H2A.Z-2 forms (Fig. 1, lanes 1–4).

Previous studies have reported that PIAS4, a SUMO E3-ligase, is required for the accumulation of SUMO1 at sites with DNA damage,^23^ raising the possibility that PIAS4 is responsible for the SUMOylation of H2A.Z-2. To address this, we next examined the physical interaction between PIAS4 and H2A.Z-2. By the immunoblotting using anti-PIAS4 antibodies, we found that PIAS4 was also contained in the purified H2A.Z-2 complex, indicating its association with H2A.Z-2. Importantly, the association of H2A.Z-2 with PIAS4 was increased by irradiation (Fig. 1, lanes 5 and 6).

**Figure 2.**
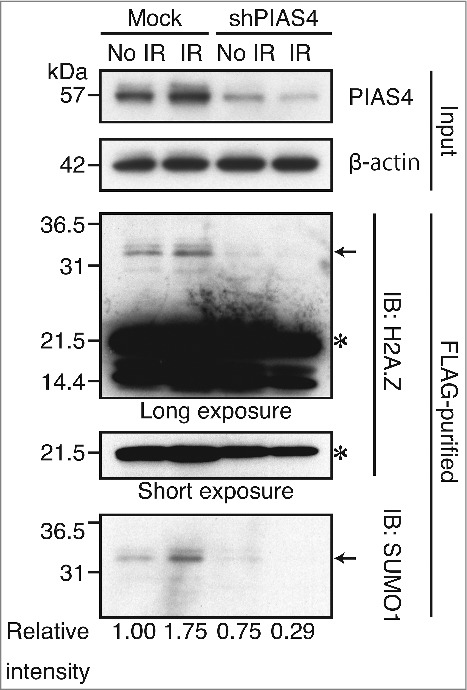
The H2A.Z-2 complex, purified from the nuclear soluble fraction of HeLa cells stably expressing mock shRNA or shPIAS4, was subjected to immunoblot analyses using anti-H2A.Z and anti-SUMO1 antibodies. The amounts of PIAS4 and control β-actin in the input materials were detected by immunoblotting with the respective antibodies. DNA damage was induced by 10 Gy IR, followed by a 10-minute recovery. The arrows indicate SUMOylated H2A.Z-2 and the asterisks indicate unmodified H2A.Z-2. SUMOylated H2A.Z-2 protein levels were calculated as relative intensity with respect to β-actin. Nuclear extracts are used as the input.

To confirm that the above-mentioned DNA damage-dependent SUMOylation of H2A.Z-2 was indeed mediated by PIAS4, we examined the effect of PIAS4 depletion on the SUMOylation of H2A.Z-2. To do so, we established HeLa cells in which PIAS4 is depleted by shRNA-mediated downregulation, and subsequently performed the immunoblotting analysis. As shown in Fig. 2, significant decreases of the SUMOylated H2A.Z-2 were detected both before and after DNA damage (indicated by arrows), indicating that PIAS4 is the E3-ligase involved in the SUMOylation of H2A.Z-2. Remarkably, the H2A.Z-2 SUMOylation after irradiation was nearly abolished by the PIAS4 depletion (relative intensity of SUMO1 from 1.75 to 0.29), suggesting that the DNA damage-induced SUMOylation of H2A.Z-2 is predominantly mediated by PIAS4 (Fig. 2).

**Figure 3.**
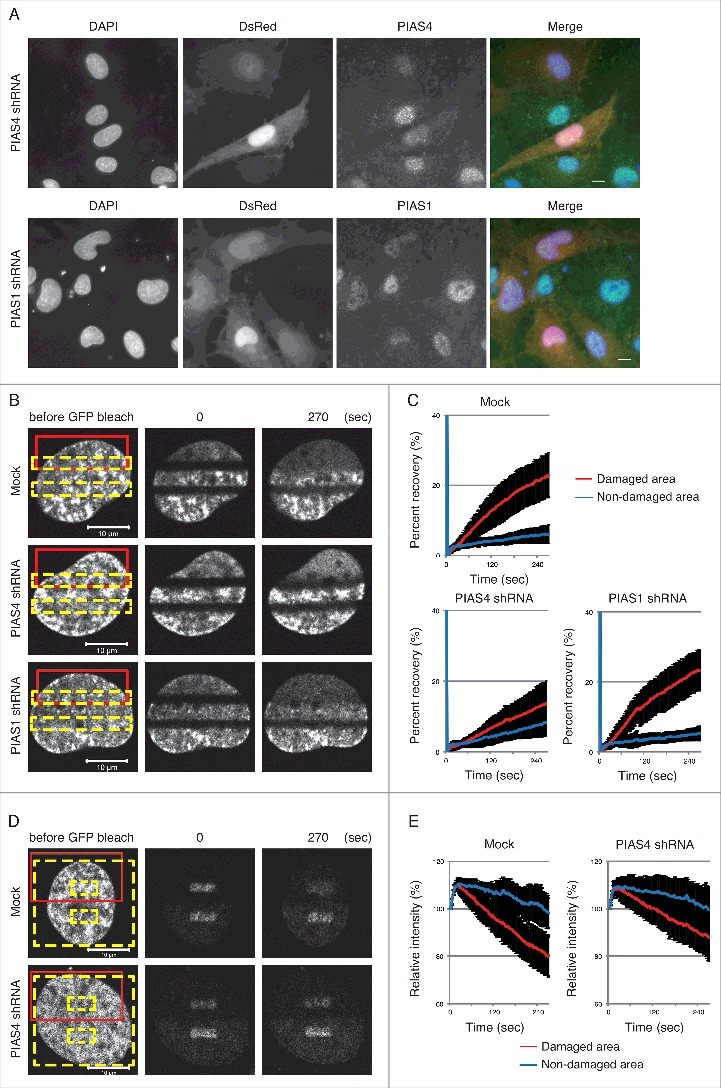
(A) Depletion of PIAS4 or PIAS1 by pSIREN-shRNA. Cells expressing pSIREN-shRNA are DsRed-positive. Endogenous PIAS4 and PIAS1 were detected by immunofluorescence staining with the respective antibodies. DsRed, PIAS4 and DNA (DAPI) are shown in red, green and blue, respectively, in the merged images. Scale bars: 10 μm. (B) FRAP analysis to monitor the incorporation of H2A.Z-2 at damage sites. GM0637 cells transiently expressing GFP-H2A.Z-2 and pSIREN-mock, PIAS4 or PIAS1 shRNA were first microirradiated (red boxes) and then photobleached (yellow boxes). (C) The fluorescence recovery of the cells in (B) was monitored as previously described.^15^ (D) Inverse FRAP analysis to monitor the eviction of H2A.Z-2 at damage sites. GM0637 cells transiently expressing GFP-H2A.Z-2 and pSIREN-mock or PIAS4 shRNA were first microirradiated (red boxes) and then photobleached (yellow boxes, excluding small interior boxes). (E) The relative intensity of the cells in (D) was monitored as previously described.^15^

We have previously shown that H2A.Z-2 is exchanged at DSB sites.^15^ To examine whether the SUMOylation of H2A.Z-2 plays a key role in the dynamics of this exchange, we performed fluorescence recovery after photobleaching (FRAP) in combination with UVA-microirradiation, using cells transiently expressing GFP-fused H2A.Z-2 together with shRNA against either PIAS4 or PIAS1 (Fig. 3A). The cells were first microirradiated (Fig. 3B, red boxes) and then photobleached (Fig. 3B, yellow boxes), to analyze the recovery of the fluorescent signal in the bleached area. The significant fluorescence recovery of the GFP-H2A.Z-2 signal was observed after microirradiation (Fig. 3C, red line), but not within the unirradiated areas, in the mock-shRNA transfected cells as reported previously (Fig. 3C, blue line).^15^ In contrast, the fluorescence recovery of the GFP-H2A.Z-2 signal after microirradiation was significantly repressed in the PIAS4 shRNA-expressing cells (fluorescence recovery in the damaged area at 270 seconds after photobleaching is 13.8% ± 6.3%, with a *P* value of <0.001 between mock shRNA or shPIAS4, and fluorescence recovery in the non-damaged area is 8.3% ± 3.8%) (Fig. 3C and D). It has been reported that another SUMO E3-ligase, PIAS1, also accumulates at DSB sites and promotes DNA damage responses.^23^ However, the PIAS1 depletion failed to repress the fluorescence recovery of the GFP-H2A.Z-2 signal at the microirradiated area. These findings suggest that PIAS4, but not PIAS1, facilitates the incorporation of H2A.Z-2 at damaged sites.

Next, we examined whether PIAS4 regulates the eviction of GFP-H2A.Z-2 from the microirradiated area, by an inverse FRAP analysis.^15^ In the inverse FRAP analysis, the cells were first microirradiated (Fig. 3D, red boxes) and then photobleached (Fig. 3D, yellow boxes, excluding small interior boxes). The loss of fluorescence from the unbleached area was monitored and quantified. Consistent with our previous report, the intensity of the remaining GFP-H2A.Z-2 fluorescent signal was decreased in the irradiated areas, but not in the unirradiated areas in the mock shRNA-expressing cells (Fig. 3D and E).^15^ The inverse FRAP analysis of the PIAS4 shRNA-expressing cells revealed that the intensity of the remaining GFP-H2A.Z-2 fluorescent signal from the unbleached area was not significantly decreased in the irradiated areas, as compared to that in the mock shRNA-expressing cells (Fig. 3D and E). These findings indicate that PIAS4 facilitates the eviction of H2A.Z-2 from damaged chromatin. Taken together with the findings obtained by the FRAP analysis, these results strongly suggest that the PIAS4 mediated-SUMOylation of H2A.Z-2 regulates the exchange of H2A.Z-2 at DNA damage sites.

## Discussion

Reorganization of damaged chromatin plays an important role in the regulation of the DNA damage response. In our previous study, we found that H2A.Z-2 is exchanged at damaged sites.^15^ In this study, we showed that the SUMO modification system positively regulates the DNA damage-dependent exchange of the histone variant H2A.Z-2 at damaged sites. We also found that H2A.Z-2 is SUMOylated by PIAS4 in a DNA damage-dependent manner. These findings suggest that the SUMO modification system facilitates the exchange of H2A.Z-2 at damaged sites.

In our previous study, we showed that H2A.Z-2 is required for the DNA damage-dependent RAD51 focus formation.^15^ RAD51, a key recombinase in HR, has a SUMO-interacting motif (SIM) that is necessary for the accumulation at sites of DNA damage, and PIAS4 is required for its accumulation at DNA damage sites.^24^ In this study, we showed that PIAS4 is also responsible for the SUMOylation of H2A.Z-2. Taken together, these findings suggest that the DNA damage-dependent SUMOylation by PIAS4 facilitates the RAD51 focus formation, through the reorganization of damaged chromatin by the exchange of H2A.Z-2.

Recent studies have revealed the role of the post-translational modifications of H2A.Z in the regulation of DNA metabolism. The acetylation of H2A.Z contributes to transcriptional activation.^26,27^ TIP60 is involved in the acetylation of H2A.Z, as well as H2A and H4.^28^ The lysine methyltransferase SETD6 monomethylates H2A.Z on lysine 7, which is involved in the negative regulation of gene expression.^29^ Monoubiquitinated H2A.Z is enriched on the inactive X chromosome, suggesting that ubiquitinated H2A.Z is associated with transcriptional silencing.^30^ In contrast to these modifications involved in gene expression, the SUMOylation of H2A.Z is required for DSB recruitment to the nuclear periphery in yeast.^22^ In our study, we demonstrated that the SUMOylation of H2A.Z in human cells is also involved in the positive regulation of DNA repair. Although the means by which the SUMOylation of H2A.Z-2 in human cells facilitates the RAD51 focus formation remain to be clarified, these findings suggest the conserved function of the SUMOylation of H2A.Z to facilitate DNA repair, from yeast to humans.

H2A.Z-1 and H2A.Z-2 differ by only three amino acids, but they are encoded by distinct nucleotide sequences.^13^ Chicken DT40 cells with either the *H2A.Z-1* or *H2A.Z-2* gene knock-out exhibit distinct alterations in gene expression and cell proliferation.^14^ The H2A.Z-2 deficiency sensitizes malignant melanoma cells to chemotherapy and targeted therapy.^11^ The nucleosomal H2A.Z-1 is more rapidly exchanged than H2A.Z-2 under normal conditions.^31^ In contrast, H2A.Z-2 exhibits higher mobility than H2A.Z-1 after DSB induction.^15^ In this study, we showed that the SUMO modification system regulated the dynamics of H2A.Z-2 at DNA damage sites. The DNA damage-induced exchange of SUMOylated H2A.Z-2 may play a role to accelerate the accumulation of the SUMO-interacting DNA repair proteins at damaged sites. Although further explorations are required to clarify the interaction between RAD51 with H2A.Z-2, the focus formation of RAD51 could be facilitated by this DNA damage-dependent exchange of SUMOylated H2A.Z-2. Interestingly, H2AX, another histone H2A variant, is also exchanged after the induction of DSBs, to allow PARP-1 accumulation at damaged sites.^32^ The exchange of histone variants H2AX and H2A.Z-2 may play an important role in DNA repair to facilitate the intra-nuclear transport of repair proteins to the damaged sites.

## Materials and methods

### Cell culture and ionizing irradiation

GM0637 cells were cultured in Dulbecco's modified Eagle's medium (Sigma-Aldrich), supplemented with 10% fetal bovine serum (Equitech-Bio). HeLa cells were cultured in Dulbecco's modified Eagle's medium supplemented with 10% fetal bovine serum and 0.2 mg/ml G418 (Gibco). For ionizing irradiation treatment, cells were irradiated with ^137^Cs γ-rays, using a Gammacell 40 system (MDS Nordion, Ottawa, Canada) at 10 Gy.

### Protein affinity purification

To prepare FLAG-HA-tagged H2A.Z-2 complex, nuclei were collected by centrifugation at 3,900 rpm for 15 minutes after a treatment with hypotonic buffer, as previously described.^33^ After resuspension of the pellet in an equal volume of sucrose buffer (0.34 M sucrose, 10 mM Tris-HCl, pH 7.3, 3 mM MgCl_2_, 100 mM MEM), 1 × sucrose buffer was added to adjust the volume to a final DNA concentration of 2 mg/ml. Micrococcal nuclease was added at 25 U/ mg DNA. The samples were incubated at 37°C for 20 minutes, and the reactions were then stopped by adding 4 mM EDTA. The samples were centrifuged at 14,000 rpm at 4°C for 30 minutes. The supernatant was used after dialysis, as the solubilized FLAG-HA-tagged H2A.Z-2-containing chromatin fraction. FLAG-HA-tagged H2A.Z-2 proteins were purified by immunoaffinity purification with an immobilized anti-FLAG antibody, and were eluted with FLAG peptide as described previously.^25^ The knockdown of PIAS4 was performed by the expression of pSuper-retro-PIAS4 by a retroviral vector. Nuclear extracts are used as the input. All buffers contained 100 mM *N*-ethylmaleimide (Sigma-Aldrich), to prevent deSUMOylation by SUMO proteases.

### Immunoblotting

Protein extracts were resolved by sodium dodecyl sulfate (SDS)–polyacrylamide gel electrophoresis and transferred to nitrocellulose membranes. The membranes were blocked with Blocking One (Nacalai Tesque, Inc.) for 60 minutes at room temperature. The primary antibodies, diluted in Phosphate Buffered Saline (PBS) with Tween® 20, were incubated with the membranes for 60 minutes at room temperature. The membranes were subsequently washed and incubated with horseradish peroxidase-conjugated secondary antibodies for 60 minutes at room temperature. Band intensities were quantified using densitometry (Image J software) and normalized to those of β-actin serving as the loading control. The intensities were calculated relative to that of the control (Mock-No IR), which were set to 1.0.

### Antibodies

Rabbit anti-H2A.Z (cat# ab4174, Abcam), rabbit anti-SUMO1 (cat# sc-9060, Santa Cruz Biotechnology), rabbit anti-PIAS1 (cat# ab32219, Abcam), rabbit anti-PIAS4 (cat# ab58416, Abcam), mouse anti-β-actin (cat# A5441, Sigma-Aldrich) and goat anti-rabbit Alexa Fluor 488 (cat# A11008, Life Technologies) were used in the experiments.

### UVA-microirradiation, FRAP and iFRAP

Imaging, microirradiation, and fluorescence recovery after photobleaching (FRAP) experiments were performed using an LSM780 confocal microscope (Carl Zeiss), with a 63 × 1.40 NA plan-apochromat objective. Cells were placed in no. 1S glass-bottom dishes (Matsunami Glass Ind., Ltd.). For microirradiation, sensitization of cells was performed by incubating the cells for 24 hours in medium containing 2.5 µM deoxyribosylthymine and 0.3 µM bromodeoxyuridine (Sigma-Aldrich) and then staining with 2 µg/ml Hoechst 33258 (Sigma-Aldrich) for 10 minutes before UVA microirradiation, as described previously.^34^ The Dulbecco's modified Eagle's medium was replaced by Leibovitz's L-15 (Gibco) containing 10% fetal bovine serum and 25 mM HEPES (Gibco), just before microirradiation. During imaging, the dishes were kept in a humidified cell culture incubator with a continuous supply of 5% CO_2_/air at 37°C (Tokai Hit). The 355-nm line of the UVA laser was used for microirradiation (six pulses at 4.43 W). The maximum power of the 488-nm Ar laser line was used for photobleaching in the FRAP analysis. For imaging, the laser was attenuated to 0.1%. All fluorescence regions except for small regions in the irradiated and unirradiated areas were bleached, and the remaining GFP fluorescence was monitored with the LSM780 confocal microscope. For the FRAP and iFRAP analysis, a prebleached image was acquired just after the induction of DSBs by UVA laser microirradiation, after which the bleaching pulse was delivered. To quantify the fluorescence recovery, single optical sections were collected at 3-s intervals for the indicated periods of time. ImageJ was used for fluorescent intensity quantification in the FRAP and iFRAP analysis. The relative intensities in the bleached area were measured and normalized by the average intensity before bleaching. The percent recovery (relative intensity) at each time point was calculated as: *P*_recovery; t_ = 100 × (*I _rel__; t_*-*I _rel__; 1.5s_*)/(1-*I _rel__; 1.5s_*), where *I _rel__; 1.5s_* was the relative intensity of the bleached area in the first image obtained after bleaching.

### Immunofluorescence microscopy

Cells were fixed with PBS containing 2% paraformaldehyde for 10 minutes at room temperature, and permeabilized with PBS containing 0.5% Triton X-100 for 10 minutes at room temperature. The cells were then incubated with antibodies in PBS containing 1% BSA, at 37°C for 30 minutes. Nuclei were stained with DAPI. The cells were mounted using Vectashield and observed on an Axioplan2 microscope with AxioCam MRm, controlled by the AxioVision software (Carl Zeiss).

### RNAi

The pSIREN-DNR-DsRed-Express vector (Clontech) was used for PIAS1 and PIAS4 RNAi. The target sequences were 5′-CGAAUGAACUUGGCAGAAA-3′ (PIAS1) and 5′-AGGCACUGGUCAAGGAGAA-3′ (PIAS4).

### Statistical analysis

Data were compared using the Student *t*-test.
